# Resilience in the Endurance Runner: The Role of Self-Regulatory Modes and Basic Psychological Needs

**DOI:** 10.3389/fpsyg.2020.558287

**Published:** 2021-01-06

**Authors:** Pierluigi Diotaiuti, Stefano Corrado, Stefania Mancone, Lavinia Falese

**Affiliations:** Department of Human, Social and Health Sciences, University of Cassino and Southern Lazio, Cassino, Italy

**Keywords:** runners, resilience, regulatory modes, basic psychological needs, SEM analysis

## Abstract

Endurance sports certainly require an important and delicate task of mental and physical reintegration from the impact of the fatigue induced by the exertion of the sport performance. The topic of the resilience of athletes has been the theme of numerous studies, however, there are few specific works on the psychological resilience of runners. Our study aimed to investigate Resilience in Endurance Runner related to the role of Self-Regulation Modes and Basic Psychological Needs. Especially, the aim of our work was presenting a model where the gratification of the Needs of Autonomy and Competence and the level of Locomotion were the predictors of the two main components of Richardson’s resilience: Homeostatic and Resilient Reintegration. The present study involved 750 endurance runners, members of the Fidal (Italian Athletics Federation). A SEM analysis was performed combining into one explanatory model the following variables: Autonomy and Competence Satisfaction, Self-Regulatory Locomotion Mode, Homeostatic and Resilient Reintegration. The model showed overall acceptable fit measurements: χ^2^ = 872.152; CFI = 0.966; TLI = 0.952; RMSEA = 0.058. Results indicated that BPNs and SRMs are predictors of the level of resilience in endurance running athletes. In particular, *Resilient Reintegration* was mainly affected by *Locomotion Mode* (β = 0.379 for *p* < 0.005), which in turn received a major influence from *Autonomy Satisfaction* (β = 0.574 for *p* < 0.001). *Homeostatic Recovery* was found to be affected by *Competence Satisfaction* (β = 0.489 for *p* < 0.001). The study pointed out the importance of supporting in endurance runners the gratification of the needs of Autonomy and Competence as key factors capable of enhancing perseverance, timely recovery and psychophysical balance.

## Introduction

Athletes that apply endurance sports are aware that their performance and activity may be affected by arbitrary elements or unforeseen events such as varying of weather conditions, mechanical failures, sudden pain or discomfort that can be associated with their mental and physical state (Meijen., 2019). Endurance performances are defined as performances during whole-body, dynamic exercise that involves continuous effort and lasts for 75 s or longer (McCormick et al., 2015). Endurance athletes differ from sprint-trained athletes and strength athletes with respect to metabolic adaptation and also with respect to the psychological mechanism highlighting their performances (Moll et al., 2018; Degens et al., 2019; Guicciardi et al., 2019).

The existing scientific literature describes the important influence of psychological variables, such as personality traits and mood state, self-efficacy, intrinsic motivation, stress and anxiety management and goal setting, on the success of endurance outcome (Raglin and Wilson, 2008; Stoeber et al., 2009; Stanley et al., 2012). Endurance athletes also require elevated levels of self-control, defined as one’s “capacity to regulate attention, emotion, and behavior in the presence of temptation” (Duckworth and Seligman, 2017) and an attitude of “never giving up,” similar to grit, more than almost any other athletic effort (Moran, 2012).

Another trait that seems to be associated with the success of sport performances of endurance athletes is resilience (Drury, 2019). Although resilience has been widely studied in different areas of psychology, only at the beginning of the 21st century the researchers begin to investigate this construct in the field of sport (Galli and Vealey, 2008; Gucciardi et al., 2011). In previous studies on a similar population, endurance runners have reported higher levels of resilient traits (such as tenacity, determination, and tolerance of negative affect) as compared to non-runners (Bebetsos and Goulimaris, 2015; Drury, 2019). This is perhaps due to the fact that endurance competitions are physical demanding and often cause stress, discomfort and physical pain, which the athletes deliberately choose to face (Raglin and Wilson, 2009; Gucciardi et al., 2017).

In a review about psychological resilience, Fletcher and Sarkar (2013) consider resilience for an athlete as the ability to accurately appraise when and how to use and optimize coping skills in order to confront stressful events and they consider it fundamental for a successful performance. Being resilient also means being able to recognize one’s limits and accept them, and have the strength to look beyond difficulties optimistically (Fletcher and Sarkar, 2013, 2016; Galli and Gonzalez, 2015). In the face of defeat and frustration resilient individuals are capable of not losing hope (Earvolino-Ramirez, 2007), and having a good self-efficacy, often positively associated to resilience, also helps them to have control over the event (Diotaiuti et al., 2017). González et al. (2019) found that athletes’ resilience was positively associated to the satisfaction of basic psychological needs (BPN) and this indirectly promotes well-being in sport (Clough et al., 1989; Reinboth et al., 2004) and contributes to predict athletes’ quality of engagement and development (González et al., 2019). It has also been seen how the satisfaction of the needs of autonomy, competence and relatedness, the three main intrinsic needs involved in self-determination, increases the internal resources related to resilience among sports individuals and having autonomous values and beliefs has a positive influence on the processes related to resilience (Sarkar and Fletcher, 2014).

The way individuals regulate their behavior to achieve goals can also influence resilience and consequently success and well-being (Garcia and Archer, 2016). Within the regulatory modes, locomotion, the individual’s capability to advance step by step until the goal is achieved (Higgins et al., 2003), seems to predict resilience (Garcia and Archer, 2016). In a recent study on the self-regulatory modes in runners that use sport trackers, it was also shown that regulatory modes can influence the attitudes toward the monitoring tool used during the runners’ performances (Diotaiuti et al., 2020). Modes of self-regulation for competitive athletes also include focus (promotion and prevention focus) and concentration (Memmert et al., 2013). Fletcher and Sarkar (2012) found that the ability to focus was an important aspect of resilience for the world’s best athletes. Numerous aspects of focus and concentration appear to be important in order to deal with pressure and adversity in various competitive sport contexts (Jones et al., 2002, 2007; Bull et al., 2005; Gucciardi et al., 2009).

The type of conceptualization currently most shared by researchers is that psychological resilience is a dynamic process that develops over time and can vary contextually and temporally (Folke, 2016). Among the various resilience models available in the literature, of particular importance is the one formulated by Richardson (2002). According to this model, the balance is continually exposed to the risk of alteration by stressful events, adversity, opportunity and other forms of change. Once the balance is compromised, the first stage of the process (destruction) begins, determined by the interaction between stressful events and protective factors, the latter supporting people to face difficulties and maintain homeostasis. In particular, the first phase is characterized by feelings such as guilt, fear, perplexity, confusion and bewilderment, emotions that can lead to a lack of self-confidence or the fear of not being able to develop the necessary skills to deal with change. After the first phase, a second phase (reintegration) begins, in which individuals decide, consciously or unconsciously, which type of reintegration to implement. Richardson’s model proposes four possibilities for this second phase: *resilient reintegration*, which refers to the coping process that determines growth, knowledge, self-understanding and development of resilient characteristics; *homeostatic reintegration*, the reintegration that leads to the initial homeostasis, in which the growth of the individual and the development of resilient characteristics is not expected; *reintegration with loss*, which happens when people are no longer motivated and give up; *dysfunctional reintegration*, which occurs when people resort to drugs, destructive behavior or other means to deal with adversity. Failure to resilient reintegration increases the likelihood that expected or unforeseen unpleasant events act as risk factors, making the person who has not developed his resilient qualities even more vulnerable.

We believe that this resilience model of Richardson can be usefully addressed in sports and specifically in the case of endurance runners. Endurance sports indeed certainly require an important and delicate task of “reintegration” for athletes, both physically and mentally (Pageaux and Lepers, 2016). From the consideration of the empirical literature cited above, in particular González et al. (2019), in which it emerged a clear predictive finding between the satisfaction of basic psychological needs and the resilience of the athlete, Fletcher and Sarkar (2012) and Garcia and Archer (2016), in which the ability to focus and to advance step by step until the goal is achieved (corresponding to the self-regulatory mode of Locomotion) were further significant predictors of resilience, we have hypothesized that in endurance runners both the gratification of basic psychological needs such as autonomy and competence and the level of locomotion can be assimilated to the protective factors of Richardson’s resilience, which help to cope with difficulties and maintain homeostasis. Our work was therefore aimed at presenting a model where the gratification of the Needs of Autonomy and Competence and the level of Locomotion were the predictors of the two main components of Richardson’s resilience: Homeostatic and Resilient Reintegration. In accordance with the results of the above mentioned literature, the model tested the adequacy of the predictive relationship between Autonomy and Competence Needs gratification and Locomotion level with the highest stage of reintegration, i.e., resilient; while the gratification of the need for recognition of Competence was placed in predictive relationship with Homeostatic Reintegration.

## Materials and Methods

### Sample Selection and Questionnaire Administration Procedures

The information needed to confirm the working hypotheses had been collected from the administration of a questionnaire to a representative sample of athletes on a regional scale. The reference target concerned runners belonging to the 146 running sports associations of the Lazio Region, members of the Fidal (Italian Athletics Federation), which accounted for about 22,000 athletes in June 2019. The sample size determination was made according to Kline (1998), who remarked that an adequate sample size for a SEM should always be ten times the amount of the parameters. Therefore, since our hypothetical model would have foreseen five factors with 21 variables, considering 26 variances and as many covariances, the number of 73 predictors to be estimated and therefore a sample of at least 730 participants was identified. Participants had been recruited by way of a preliminary connection with the presidents of the running sports associations, who ensured the dissemination of the questionnaire to the members of theirs, through the forwarding of a contact in which the goals as well as function of the study had been mentioned as well as in which, subjects had been invited to get into a specific link found in the very same notice after which fill in and post the answers telematically and digitally. Participants had been sure anonymity and also the usage of information in aggregate type for research purposes only. The typical length of time for the compilation was approximately 20 min. A total of 3,000 contact emails were sent. As far as the dropping ratio is concerned, 48 participants resulted to drop out after starting to filling in, so 750 completed questionnaires were finally collected. As can be noticed in Table 1, where the socio-demographic characteristics of the sample are reported, participants (86.9% males) were aged between 20 and 65 years (*M* = 42.58; SD = 7.81); the average number of competitions in which they participated annually was 13.70 races (SD = 6.65), and overall the 42.7% of the sample declared to have competed in at least one marathon in the last three years.

**TABLE 1:** Socio-demographic characteristic of the sample.

		*N*	(%)
Gender	Males	652	86.9
	Females	98	13.1
Age	20–36 years	166	22.2
	37–45 years	251	33.4
	46–49 years	176	23.5
	50–65 years	157	20.9
Work	Employed	526	70.2
	self-employed	138	18.4
	Students	55	7.3
	Unemployed	31	4.1
Experience	1–3 years	194	25.7
	4–5 years	227	30.3
	6–10 years	198	26.5
	11–34 years	131	17.5
Favorite Speciality	10 km race	351	46.7
	Half marathon	232	31.0
	Marathon	137	18.3
	Cross-country race	30	4.0
Motivation to Run	Fitness	213	28.4
	Antistress	163	21.7
	Example of friends	137	18.2
	Desire to exceed own limits	164	21.9
	Suitable and natural for him/her	73	9.8

### Tools

To be able to gather the information required to carry out the study, a questionnaire was built up and articulated into the coming sections: (1) socio demographic info: gender; age; (2) specifications as a runner: sports specialty, i.e., ten km, half marathon, marathon, cross country; years of expertise in competitive running; (3) opinion on the preponderant element for a good competitive preparation: option between mindful preparation, intense training, constant monitoring; (4) psychometric measurements: (a) *Resilient Reintegration*, subscale of the *Resilience Process Questionnaire* (RPQ; Richardson, 2002; Italian validation: Laudadio et al., 2011), refers to the coping process that determines growth, knowledge, and understanding of oneself and the development of resilient features. It consists of five items depending on a five points Likert scale (one = disagree; five = completely agree), and Cronbach’s alpha in this particular study was 0.831; (b) *Homeostatic Reintegration*, subscale of the *Resilience Process Questionnaire* (RPQ; Richardson, 2002; Italian validation: Laudadio et al., 2011), characteristic of subjects that, in the face of stress or trauma, attempt to recover the state of equilibrium just before the event; five points Likert scale (one = disagree; five = completely agree), with a Cronbach’s alpha 0.878; (c) *Locomotion Mode*, subscale of the *Self-Regulatory-Modes Scale* (RMS; Higgins et al., 2003; Italian validation: Pierro et al., 2006), is the part of the self adjusting system devoted to managing the motion by state as well as the maintenance of its to attain an objective in an easy method and with no delays or distractions. It is made up of twelve items, six point Likert (from one = totally disagree to six = absolutely agree) and in this particular study Cronbach’s alpha was 0.712; (d) *Autonomy* satisfaction (four items; e.g., “I feel a sense of choice and freedom in the things I undertake”) and *Competence* satisfaction (four items; e.g., “I feel confident that I can do things well”), both subscales of the *Basic Psychological Need Satisfaction Scale* (BPNSS, Chen et al., 2015; Italian validation: Costa et al., 2018) that measures positive experiential state that occurs when people perceive their psychological basic need satisfied in their life in general. Participants responded on a 5-point Likert scale ranging from 1 “completely disagree” to 5 “completely agree” and Cronbach’s alpha resulted, respectively, 0.73 and 0.74.

## Statistical Analysis

The data were processed using the statistical software SPSS version 22. The main analyses performed were: descriptive statistics to illustrate socio-demographic information, specifications as a runner; Pearson bivariate correlations for all main measures (*Autonomy* and *Competence Gratification*, *Locomotion Mode*, *Resilient*, and *Homeostatic Reintegration*) significant at *p* < 0.005 and at *p* < 0.001, 2-tailed); Cronbach’s alpha as scale reliability coefficient; SEM analysis to test predictors’ effects on Homeostatic and Resilient Reintegration of runners. To test the adequacy of the model, as also suggested by Teo (2010), the following fit indices were considered: (1) the chi-square; (2) CFI (Comparative Fit Index); (3) TLI (Tucker Lewis Index); (4) RMSEA (Root-Mean-Square Error of Approximation), with CFI and TLI > 0.95 and RMSEA < 0.06 as excellent model fit indicators (Yu, 2002).

## Results

### Preparatory Data Analysis

Outliers were assessed by inspection of a boxplot, normality was assessed using Shapiro-Wilk’s and homogeneity of variances was assessed by Levene’s test. There were no outliers, residuals were normally distributed (*p* > 0.05) and there was homogeneity of variances (*p* > 0.05). The preliminary verifications of the regression assumptions excluded the presence of multivariate outliers. Mardia’s multivariate kurtosis index (15.85) was in fact below the critical value [*p* (*p* + 2) = 99]; therefore, the relationship between the variables can be considered substantially linear. Low co-linearity was indicated by the low VIF values (Variance Inflation Factor) <2 and high tolerance values > 0.60. For verification of the assumptions on the residuals, the average between the standardized and raw residuals was equal to 0; the Durbin–Watson test had a value of 1.97 and was therefore indicative of the absence of autocorrelation.

### Measurement and Structural Model

Table 2 below reports correlations between the variables of the study. As can be noted, several significant associations have emerged: first of all between Autonomy Satisfaction and Locomotion (0.353**); between Locomotion and Resilient Reintegration (0.271**); between Competence Satisfaction and Autonomy Satisfaction (0.342**); finally between Competence Satisfaction and Homeostatic Reintegration (0.374**).

**TABLE 2:** Pearson’s bivariate correlations.

	AUT	COMP	RES	HOM
COMP	0.342**			
RER	0.411**	0.291**		
HOR	0.312**	0.374**	0.325**	
LOC	0.353**	0.340**	0.271**	0.410**

AUT, Autonomy Satisfaction; COMP, Competence Satisfaction; RES, Resilient Reintegration; HOM, Homeostatic Reintegration; LOC, Locomotion Mode. *N*, 750; **Correlation is significant at the 0.01 level (2-tailed).

**FIGURE 1:**
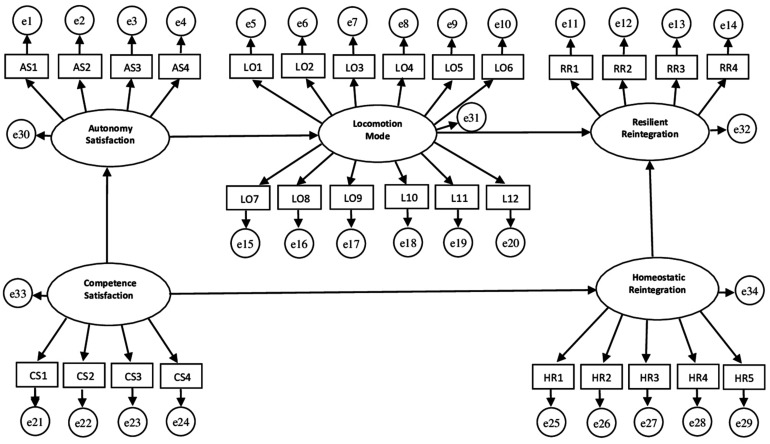
Assumed model.

In light of the associations that have emerged and taking into account the results of empirical literature previously mentioned, we aimed to test the adequacy of a predictive model of Homeostatic and Resilient Reintegration of the endurance runner, in which (as shown in Figure 1) the predictor of Homeostatic Reintegration was identified in the satisfaction of Competence, while in the mode of Locomotion, in turn enhanced by the satisfaction of the Autonomy of the athlete, the major predictor of Resilient Reintegration.

**FIGURE 2:**
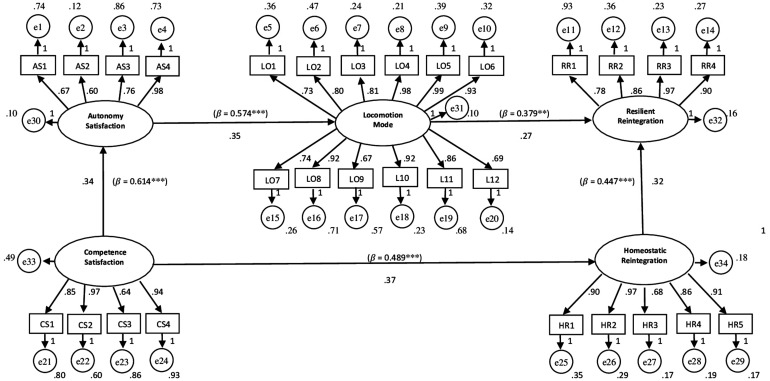
Structural equation model (SEM).

As suggested by Mackinnon (2008), before conducting a SEM analysis a separate Confirmatory Factor Analysis (CFA) was performed for each of the following constructs: Autonomy Satisfaction (χ^2^ = 10.444; CFI = 0.965; TLI = 0.961; RMSEA = 0.052), Competence Satisfaction (χ^2^ = 8.618; CFI = 0.952; TLI = 0.953; RMSEA = 0.059), Locomotion Mode (χ^2^ = 10.240; CFI = 0.962; TLI = 0.957; RMSEA = 0.053), Homeostatic Reintegration (χ^2^ = 15.743; CFI = 0.967; TLI = 0.953; RMSEA = 0.056), Resilient Reintegration (χ^2^ = 16.570; CFI = 0.969; TLI = 0.954; RMSEA = 0.058). Subsequently the general SEM analysis was carried out. The tested model showed overall acceptable fit measurements: χ^2^ = 872.152; CFI = 0.966; TLI = 0.952; RMSEA = 0.058. The whole model with both predictions and correlations between constructs is displayed in Figure 2, where it is shown that *Resilient Reintegration* was mainly affected by *Locomotion Mode* (standardized estimate of the regression weight of 0.379 for *p* < 0.005), which in turn received a major influence precisely from *Autonomy Satisfaction* (standardized estimate of the regression weight of 0.574 for *p* < 0.001). *Homeostatic Reintegration* was found to be affected by *Competence Satisfaction* (standardized estimate of the regression weight of 0.489 for *p* < 0.001). The latter also showed a main influence on *Autonomy Satisfaction* (standardized estimate of the regression weight of 0.614 for *p* < 0.001). The last influential relationship identified by the model was that exerted by *Homeostatic Reintegration* on *Resilient Reintegration* (standardized estimate of the regression weight of 0.447 for *p* < 0.001). Finally Table 3 below summarizes the Maximum Likelihood Estimates and the corresponding Standardized Regression Weights Estimates between model’s constructs. Also from this table it can be observed that while the satisfaction of the need of Competence constitutes the factor that influences the Homeostatic Reintegration, this last one together with the Locomotion Mode are predictive variables of the higher level of Reintegration that according to the model of Richardson is the Resilient one.

**TABLE 3:** Maximum Likelihood Estimates and Standardized Weight Estimates.

Label		Label	MLE	S.E.	C.R.	*P*	SWE
Competence satisfaction	→	Autonomy satisfaction	0.531	0.152	4.070	***	0.614
Autonomy satisfaction	→	Locomotion mode	0.594	0.160	4.242	***	0.574
Competence satisfaction	→	Homeostatic reintegration	0.470	0.113	5.124	***	0.489
Locomotion mode	→	Resilient reintegration	0.363	0.140	3.167	0.001	0.379
Homeostatic reintegration	→	Resilient reintegration	0.336	0.121	3.573	***	0.447

MLE, Maximum Likelihood Estimates; SWE, Standardized Regression Weight Estimates. ***p < 0.001.

## Discussion

The results of the SEM analysis confirmed the adequacy of the hypothesized model. A predictive relationship between the gratification of the Basic Psychological Needs (BPN), Regulatory Modes of the Self (RMS) and the resilience of the endurance athlete was found. More specifically, it was found among RMS significant implication of the Locomotion Mode and among BPN the implication of the Satisfaction of Competence and Autonomy on athletes’ psychological recovery (resilient or homeostatic).

With regard to Resilient Reintegration [identified by Richardson et al. (1990), Richardson (2002) as the genuine process of growth, knowledge, self-awareness, and resilient capacity building], an influential predictor has been found in the Locomotion Mode. This evidence can be explained by the energies and internal resources of individuals who exhibit high levels of locomotion as described by the previous scientific literature (see Kruglanski et al., 2000; Pierro et al., 2006; Scholer and Higgins, 2012; Lucidi et al., 2016).

According to García-Secades et al. (2017), when the recovery resources start to be much less than the requirements posed by the hard conditions, a bad cycle could start for the athlete which might result in a rupture of homeostasis, pressing him/her to a continuous increase in anxiety levels. Full recovery is therefore allowed by a functional synergy of the relational component with the internal component of orientation and focus on the objective. This result is consistent with previous studies that have brought back to the internal motivational component the athlete’s resilience and perseverance in moments of greatest difficulty and tension (Martin et al., 2015; Hill et al., 2018; Zeiger and Zeiger, 2018). Our results are also consistent with those of García-Secades et al. (2017) that found Locomotion as significant element of empowerment and predictor of positive affect that facilitate the resilience process.

As far as the Homeostatic Reintegration is concerned, as ability to return to the situation before the challenge and to the condition of psychophysical balance, Competence Satisfaction has been found among the BPN to be the influential predictor. Psychological Needs satisfaction (as that of competence) has been discovered to nurture in the sports various elements of inspiration, for example self determined regulation (Edmunds et al., 2006), intrinsic motivation as well as flow (Schüler et al., 2010), exercise behavior (Wilson et al., 2003) and well being (Reinboth and Duda, 2006; Adie et al., 2012). The need for competence in runners refers to the notion of mastering one’s contexts, experiencing effectiveness, and controlling and achieving desired outcomes.

Specifically, López-Walle et al. (2012) tested a model with Mexican athletes where the satisfaction of the BPNs positively predicted both the life satisfaction and vitality of the person, and this seems to be consistent with our study which found that the satisfaction of the need for competence has a significant effect on the recovery of the athlete’s psychophysical balance.

Autonomy Satisfaction, another BPN, has really been found to raise perceived competence (Chiviacowsky, 2014) or efficacy (Wulf et al., 2014). Opportunities for choice improve expectations for experience that is positive as well as outcomes in sport performances (Lemos et al., 2017). Our model, compared to the above mentioned literature, presents instead an inverse relationship of the effect between autonomy and competence; that is, the gratification of competence influences autonomy. As the athlete feels more competent, the need for autonomy increases and this feeds his resilient reintegration capacity through a decisive impulse given to the athlete’s goal orientation (Locomotion). In Iwatsuki et al. (2019) contribution has been expressly addressed the theme of increasing the efficiency of running by autonomy.

To the best of our knowledge, literature has not linked the gratification of the need for autonomy to the resilient abilities of the athlete. From our results instead, the indirect effect on resilient recovery (through enhancement of the Locomotion mode) was evident. Therefore by ensuring greater autonomy support climates and greater engagement as well as autonomous motivations, athletes might be a lot more shielded from stressors boosting their optimal mental functioning and resilience.

Finally the tested model hinted that Homeostatic Reintegration contribute with a positive effect on Resilient Reintegration. This effect can be traced back to a reason of experience and learning: having repeatedly and brilliantly overcome crises and particularly difficult moments over time supposedly favors a different attitude in the athlete in the face of difficulty as a further opportunity for growth and experience, rather than a threat and a risk.

From the results described so far, the hypothesis of the work, namely that the dimensions of resilience had both BPN and SRM components as predictors, has found verification in the analyses conducted.

Some limitations can however be detected in this study such as the specificity of the sample (endurance athletes). Their generalization would require an extension of the study to athletes of other sports disciplines (where, for example, the predominant component is speed, power, dexterity or targeting), both individual and team sports. Due to the specificity of the sample and after a first investigative analysis we have decided not to consider some variables such as the BPN of relatedness and this can also be considered a limit since there are studies proving that social relatedness is important for individual athletes as well (Schüler et al., 2014). A further limitation is the adoption of a multidimensional and general resilience model, which does not currently have a specific adaptation for sports contexts; moreover, on the BPNSFS scale only the section related to the measure of the gratification of Basic Needs has been administered, so an extension of the study should also include a comparison with the measures of frustration of perceived needs. In the perspective of a systemic deepening of resilience resources, not only individual athletes but also coaches and their technical staff should be involved in the study. The innovative contribution of the work lies in having firstly made explicit in a comprehensive model the influence of BPNs and Locomotion’s Self Regulating Mode on the resilient capacity of the endurance runner.

Except for dispositions and attitudes consolidated in the person, which determine a more impulsive and proactive or controlled and reflective nature, the gratifications of the athlete’s deep needs and the modeling by interaction with the context significantly interact with the regulatory attitude of the person, by virtue of which, in difficulty or stress, the athlete will propose with greater or lesser momentum and perseverance in the effort to overcome or recover from the crisis.

## Highlights of the Work

Resilience model of Richardson can be usefully addressed in the case of endurance runners as they have to face important and delicate reintegration tasks (both physically and mentally). BPNs and SRMs can variously affect the resilience of the athlete. Satisfaction of the Needs for Competence and Autonomy have shown to have positive effects, together with the SRM of Locomotion, on the Reintegration (Homeostatic and Resilient) of the endurance runner.

## Applications of the Findings for Athletes and Coaches

Some motivational aspects that improve the resilient ability of the athlete are directly related to the didactic situations, i.e., how the coach plans and organizes the learning and technical improvement activities. It is important to keep always in mind that the desire to perceive and demonstrate competence motivates the athlete, prompting him/her to systematically engage in increasingly strenuous training. Athletes feel motivated (and act accordingly) when they think they have the competence to respond to the demands of the task and feel they can work independently. Some methodological indications for the use in question, as a motivating factor for training and especially for competition situations, are the following: individualize the objectives; identify significant objectives; specify medium and long term; prioritize performance and not results. However, athletes should be encouraged to feel responsible for their performance, both positive and negative. In this way you help to convey the feeling of being able to control personal performance through specific and meaningful training.

## Conclusion

Overall the study showed through a SEM analysis that gratification of the needs of Competence and Autonomy and the Self-regulatory Locomotion Mode are predictors of the level of psychological recovery (Homeostatic and Resilient) among endurance runners. The adoption of the multidimensional construct of Resilience by Richardson (2002) allowed differentiating the effects of recovery factors that have been included in the model. The study pointed out the importance of supporting in endurance runners the gratification of the needs of Autonomy and Competence as key factors capable of enhancing perseverance, timely recovery and psychophysical balance. Such results could be significant also in a preventive and empowerment perspective for the resilience of the endurance athlete, encouraging the development of interventions aimed at improving the level of awareness of past experiences and the development of greater involvement of the athlete in the choice of strategies for preparation and management of the competition.

## Data Availability Statement

The raw data supporting the conclusions of this article will be made available by the authors, without undue reservation.

## Ethics Statement

The studies involving human participants were reviewed and approved by Institutional Review Board of the University of Cassino and Southern Lazio. The patients/participants provided their written informed consent to participate in this study.

## Author Contributions

PD, SC, and SM designed the study. PD, SC, LF, and SM analyzed the data and discussed the results. PD, LF, and SM drafted the manuscript. SC and SM revised the manuscript. All authors approved the final manuscript. Finally, the authors have agreed to be accountable for all aspects of the manuscript in ensuring that questions related to the accuracy or integrity of any part of it are appropriately investigated and resolved.

## Conflict of Interest

The authors declare that the research was conducted in the absence of any commercial or financial relationships that could be construed as a potential conflict of interest.
